# Developing research skills in medical students online using an active research study

**DOI:** 10.1186/s12909-023-04781-5

**Published:** 2023-10-26

**Authors:** Aziz U. R. Asghar, Murat Aksoy, Alison I. Graham, Heidi A. Baseler

**Affiliations:** 1 Centre for Anatomical and Human Sciences, Hull York Medical School, University of Hull, Hull, HU6 7RX UK; 2 Experimental Medicine and Biomedicine, Hull York Medical School, University of Hull, Hull, HU6 7RX UK; 3 York Biomedical Research Institute, University of York, York, YO10 5DD UK; 4 Health Professions Education Unit, Hull York Medical School, University of York, York, YO10 5DD UK; 5 Department of Psychology, University of York, York, YO10 5DD UK

**Keywords:** Student engagement, Research skills, Online teaching, Teaching format, Medical school, Research study

## Abstract

**Background:**

Developing research skills and scholarship are key components of medical education. The COVID-19 pandemic necessitated that all teaching be delivered online. We introduced an approach to small group teaching in the academic year 2020–2021 online which involved students in an active (ongoing) research study to develop their research skills.

**Methods:**

We acquired student feedback to evaluate their perspectives quantitatively on development of research and scholarship skills, teaching content and format, and tutor performance using this teaching approach. In addition, we captured free text responses from both students and tutors on the positives and negatives of our course, and their suggested improvements. We also compared summative assessment marks for the online/active research course (2020–2021) with those obtained from previous (2017–2019) and subsequent (2021–2023) teaching sessions.

**Results:**

Students were largely positive about most aspects of the online course utilising an active research study (*n* = 13). Students agreed that they were able to acquire research skills, particularly related to data analysis, transferable skills, and giving scientific presentations. A one-way ANOVA revealed no significant difference for assessment marks across all five teaching years (two years prior and two years following the online/active research course), indicating that the course achieved the learning outcomes. Students enjoyed the convenience of online teaching and the availability of course resources, but least liked the lack of in-person interaction and laboratory training. Tutors enjoyed the collaborative aspects of online teaching, but least liked the lack of face-to-face interactions with students.

**Conclusions:**

Our study demonstrates that delivering online teaching which involves students in active research engages and motivates them to develop their research and scholarship skills. We recommend that educators consider incorporating a current research study in their undergraduate courses as this can enhance the student learning experience as well as the research project itself.

## Background

The General Medical Council in the United Kingdom requires that medical students achieve ‘*Professional Knowledge’* learning outcomes related to *‘Clinical research and scholarship’* [1]. The outcomes stipulate that *‘… newly qualified doctors must be able to apply scientific method and approaches to medical research and integrate these with a range of sources of information used to make decisions for care.*’ Specifically, they must be able to: ‘*Interpret and communicate research evidence in a meaningful way …*’; ‘*Describe the role and value of … quantitative methodological approaches to scientific enquiry*’; ‘*Interpret common statistical tests used in medical research …*’; ‘*Critically appraise a range of research information … as reported in the medical and scientific literature*’; and ‘*Describe basic principles and ethical implications of research governance …*’. One way the Hull York Medical School addresses this requirement is via its compulsory Scholarship and Special Interest Programme (SSIP), equivalent to the Student-Selected Component (SSC) in other medical schools in the United Kingdom.

Evidence for how best to teach research methods to undergraduate medicine students is limited, although there have been attempts to review best practice in this area [2]. Training in research skills can be integrated into the main curriculum and/or be available through extra-curricular components [3]. Transferrable research skills such as critical thinking and problem solving can be integrated into the main curriculum relatively easily. However, given the time and resource requirements needed for more authentic research experiences (for example, extended research projects), it may not be possible to offer these to all students. Laidlaw and colleagues suggest that student-selected components are a key space within the medical curriculum in which research skills can be developed [3].

The SSIP allows all undergraduate medical students to develop their academic research and scholarship skills and is led by tutors who are researchers and experts in their fields. Students select and study a specific area of interest in depth within fields including neuroscience, immunology, pharmacology, nutrition, cancer, psychiatry, palliative care, public health, and health inequalities. At the Hull York Medical School, all students undertake an SSIP module in both years of Phase I (Years 1 and 2) and once in Phase II (Years 3 and 4). The SSIP discussed in this paper was aimed at Year 1 students. SSIP teaching sits alongside prescribed parts of the curriculum which includes lectures, small-group tutorials using problem-based learning, clinical skills, and placements.

The Hull York Medical School is a five-year undergraduate medical programme with an annual intake of ~ 250 students per year. The academic year is divided into three terms. As part of the current SSIP in Term 1, Year 1 students undertake a series of whole-cohort sessions on a variety of general research-related skills which provides a grounding for the discipline-specific SSIP content in Terms 2 and 3. During Term 1, they also submit their preferences from the module choices available. Students are then allocated to one of their preferred modules which they study in Terms 2 and 3. The format of individual modules can vary but must meet the following learning outcomes: 1) introduce all students to the scientific method and different approaches to research; 2) provide the opportunity for students to develop as a scholar, scientist and practitioner; 3) promote the skills and attitudes required for in-depth study; 4) promote skills relevant to the doctor as a professional, including pedagogical skills.

For medical students in Year 1, the content of the current SSIP (from 2019 onwards) in Terms 2 and 3 is delivered to groups of students by staff based within academic research centres in the Hull York Medical School. Term 2 SSIP consists of six hours of teaching which takes place over eight consecutive weeks. Term 3 SSIP consists of six hours of teaching over nine consecutive working days. Students are expected to spend 100 h on the SSIP in total, and non-timetabled time is used for self-directed learning. Prior to the COVID-19 pandemic, the SSIP modules in neuroscience consisted of small group teaching delivered in person and included laboratory-based practical sessions. Laboratory practical sessions have been demonstrated to play a vital role in science education [4–6]. The purpose of our in-person laboratory sessions was to provide students with hands-on experience of neuroscience techniques with the aim of enhancing their understanding of neuroscientific concepts. Tutors acted as expert instructors, consultants to whom students could ask questions, and facilitated group interactions [7, 8].

The COVID-19 pandemic necessitated the rapid move to an online teaching format in higher education institutions, a particular challenge for laboratory-based teaching [9–11]. Consequently, the pandemic prevented the delivery of our SSIP teaching in person for the 2020–2021 academic year. This posed a challenge for course tutors, as the replacement SSIP teaching had to enable students to successfully develop their research and scholarship skills entirely online. An innovative option was to involve the SSIP students in an active research study as a means of delivering their research and scholarship learning outcomes online. We define an ‘active research study’ as an ongoing research investigation in which data are collected and analysed concurrently while the course is being taught. This allows undergraduate students to observe the research process contemporaneously and gives them the opportunity to be involved in data collection and analysis.

Concurrent with SSIP teaching, the authors (AURA, MA, HAB) were researching the effects of COVID-19 on memory function using an online survey and memory quiz, the ‘COVID-19 Online Rapid Objective Neuro-Memory Assessment’ (CORONA) study [12]. We decided to utilise this investigation for our SSIP teaching. There are potential advantages of using an active research study for our SSIP teaching for both the students and to the research study. The primary advantage for the students would be in enabling them to gain research and scholarship skills in the absence of an in-person laboratory setting. By using an active research study, we hypothesise that students might find this more engaging and exciting than a standard practical exercise where the outcomes are known. Additionally, students could acquire skills and experience in the participant recruitment process. There could also be secondary advantages to the research study itself. For example, as students distributed the survey/memory quiz to their networks, there could be wider survey distribution, thereby increasing the size and demographic breadth of the study sample. Moreover, given the rapid output of COVID-19 research publications at that time, having multiple students engaged in literature searches enabled the timely identification of relevant literature.

The aim of this study was to explore student perceptions of an online SSIP course which involved them in an active research study. Within this context, we used a questionnaire to ask students whether the course developed their research and scholarship skills, and to evaluate the teaching content and format, as well as tutor performance. We captured and analysed student and tutor reflections on the positives and negatives of the online SSIP and possible improvements. To evaluate objectively whether learning outcomes were met successfully in the online/active research study SSIP course (2020–2021), we compared the student SSIP assessment marks across five years which included two years pre-pandemic and two subsequent years. We predicted that using an active research study to deliver SSIP teaching would interest, engage, and motivate the students while meeting the learning outcomes.

## Methods

### SSIP in-person teaching prior to COVID-19 (2017–2019)

Prior to the COVID-19 pandemic, the SSIP in neuroscience consisted of mandatory face-to-face tutorials and laboratory-based sessions delivered by three different tutors. Face-to-face tutorials (~ eight Year 1 students per tutor group) consisted of introduction to research methods, ethics, scientific oral/poster presentation and writing skills. Neuroscience-related practical sessions covered a range of topics, including clinical vision assessment and magnetic resonance imaging, recordings of neuronal oscillations and human electroencephalography. In all practical sessions, students gained live, in-person experience in experimental research design and set-up, data acquisition, analysis, visualisation, and interpretation. Additional supporting resources were provided online using the virtual learning environment (VLE), including timetables, research articles and relevant videos. Summative assessments consisted of scientific essays, posters and oral presentations based on their reading of the background literature and practical work.

### SSIP online teaching during COVID-19 (2020–2021)

During the COVID-19 pandemic, the online SSIP content was designed to meet the same learning outcomes set out by the General Medical Council as in previous years (under ‘Clinical research and scholarship’) [1]*.* SSIP teaching sessions were redesigned and delivered completely online and synchronously using video conferencing via Microsoft Teams, and again, attendance was mandatory. In Term 2, teaching sessions started in January 2021 which coincided with the third national lockdown in England, UK [13]. During the COVID-19 pandemic, Year 1 students undertaking the SSIP had no prior experience with in-person teaching in the medical school. The students were based in two geographical locations (eight students based in Hull and seven based in York) but tutors and their respective student groups were combined, and all eight teaching sessions across Terms 2 and 3 were delivered synchronously online by all three tutors together. Online tutorial sessions covered an introduction to the CORONA research study, research ethics, questionnaire distribution, research methods, data analysis and basic statistics, scientific oral/poster presentation and writing skills. The design of the online teaching sessions drew on best practice in online learning and teaching [8, 14]. The first teaching session in Term 2 included icebreaker exercises to engage with students and to replicate the informal environment of in-person small-group sessions. At the start of every online session, tutors encouraged all students to turn on their video cameras and ask questions to facilitate engagement and interaction. Students and tutors could interact via onscreen cameras and the ‘chat’ function in Microsoft Teams to allow students to give immediate feedback, provide reactions (e.g., ‘raised hand’), ask questions, and share their ideas. In one SSIP teaching session, we invited the clinicians involved in the CORONA research study to give their perspectives during an open discussion with the students. Following each online session, tutors had a debriefing session with each other reflecting on what went well and any areas of improvement.

Supporting resources were provided online on Microsoft Teams and the VLE, including timetables, research articles and relevant videos (for example, how to perform statistical tests in Microsoft Excel). Resources available to students in the online/active research course were therefore broadly equivalent to those provided in other years, although the scientific references provided were necessarily different due to the change in research topic. Students gave a formative scientific oral presentation online based on their reading of the background literature and were given written feedback from tutors. The students aided in the distribution of the CORONA survey and memory quiz during the period of the SSIP. Each student was given an individual research hypothesis/data associated with the CORONA study and undertook data analysis and interpretation to address the hypothesis. The summative assessment comprised a written scientific report. Tutors offered one-to-one online sessions to provide data analysis support, and separate sessions giving feedback on draft reports.

### Ethics and consent

The study was carried out in conformity with the principles outlined in the Declaration of Helsinki, and local ethical approval was given by the Hull York Medical School Ethics Committee (Reference 20 62). All participants were adults aged 18 years old or older and consisted of undergraduate medical students at the Hull York Medical School and their lecturers/tutors. Only participants who gave their active digital written informed consent were allowed to complete the questionnaire. As part of consenting, we informed participants that the questionnaire was voluntary and anonymous. Moreover, it was stated on the consent page of the questionnaire that taking part or not taking part would not in any way affect the SSIP assessment marks. All data collected were non-identifiable.

### Questionnaire design and dissemination

The online questionnaire was delivered using the Qualtrics platform accessed via a University of York license (Qualtrics, Provo, UT). The questionnaire was accessible via a Uniform Resource Locator (URL). The questionnaire required responses to 19 statements covering four categories: tutor performance (six statements), student skills (four statements), teaching content (four statements) and teaching format (five statements). Questionnaire statements were displayed one question at a time on the screen. Participants were instructed to indicate how much they agreed or disagreed with each statement using a slider scale. Participants were required to drag a circle (initially located at the halfway point) along a horizontal line to their selected point between two opposite labels at either end, ‘Strongly disagree’ (left) and ‘Strongly agree’ (right). There were no numerical labels on the horizontal line. This allowed respondents access to the full range of points between these two labels on the slider scale. Next, they were asked the following four open-ended free text questions to gather further details on student perceptions of the online SSIP and teaching preferences: ‘*What did you enjoy most about the SSIP being taught online?’; ‘What did you least like about the SSIP being taught online?’;‘What could be done to improve the SSIP being taught in an online format?’; ‘Which aspects of the SSIP would you prefer to be taught online and which aspects would you prefer to be taught in person?*’. Questions were created by AURA, MA and HAB based on previous literature [15–17] and other similar feedback questionnaires used within the Hull York Medical School and then reviewed by all of the authors including AIG who has experience in assessment and feedback research.

The web link to the SSIP feedback questionnaire was disseminated to all 15 Year 1 students on the neuroscience SSIP course. The questionnaire was issued on the last day of the SSIP course, one month prior to the release of the SSIP assessment marks. This was to ensure student response accuracy and avoid recall bias, and also bias based on assessment outcomes. All three SSIP tutors completed only the free text sections of the questionnaire.

### Data analysis

The responses to the questionnaire were exported from Qualtrics to Microsoft Excel (version 2210, Microsoft Corp., Redmond, WA, USA). Although not visible to the participants, the response outputs from each of the 19 slider scale statements ranged from 0 to 100 arbitrary numerical units (resolution of 1 unit), where 0 represented ‘Strongly disagree’, and 100 represented ‘Strongly agree’. Means, standard error of the means and ranges were computed across 13 respondents using Excel.

Individual student and tutor free text responses were categorised and analysed using a six-step thematic analysis as described by Braun and Clarke [18] and Kiger and Varpio [19]. Two authors (AURA, HAB) used an inductive approach to collaborative coding [20] where codes were developed whilst working through the data set and there were no preconceptions about themes. We first highlighted free text responses for key words and phrases using Microsoft Word. Next, we looked for patterns and shared meanings in the highlighted key words and phrases, and then grouped them into themes. We counted the number of student responses within each theme and calculated the frequency as a percentage of the total number of students (*n* = 13). All tutor responses were included (*n* = 3). Given the relatively small number of student and staff participants, we thought it appropriate and important to report and consider all the viewpoints.

For the neuroscience SSIP for the period 2017–2019, marks were derived from summative assessments of essays in Term 1, posters in Term 2, and oral presentations in Term 3. From academic year 2019–2020 and for all subsequent years to date, the assessment format of the SSIP was changed by the Hull York Medical School, whereby the SSIP course marks were derived from a single summative assessment of a written scientific report/essay in Term 3. However, summative assessments were suspended in the 2019–2020 academic year due to the COVID-19 pandemic. For the SSIP teaching in the years 2017–2019, the marks for Terms 1, 2 and 3 were averaged to produce a single summative mark for each student. Importantly, assessments for all years (2017–2023) were evaluated considering the same elements related to scientific background, data analysis/visualisation, and interpretation of the results in the context of the published literature. The same marking scale and rubric were used for SSIP assessments across all years: 1 = Fail (*Failed to meet many of the intended learning outcomes; work was deficient in critical aspects and demonstrated significant lack of understanding; lacked a secure basis in relevant facts and analysis; lacked a good structure.*), 2 = Pass (*Achieved the intended learning outcomes; used a sufficient range of evidence and displayed a good grasp of analytical issues and concepts; produced well-structured work.*), and 3 = Excellent (*Achieved all intended learning outcomes; used a comprehensive range of relevant materials and analyses; showed in-depth understanding of all key issues and concepts and clear evidence of critical and synthetic skills.*). A one-way ANOVA was used to compare mean summative marks across the SSIP teaching years 2017–2023, except the year 2019–2020 when no student summative assessments took place.

## Results

### Student *quantitative* perceptions to questionnaire statements

**Fig. 1:**
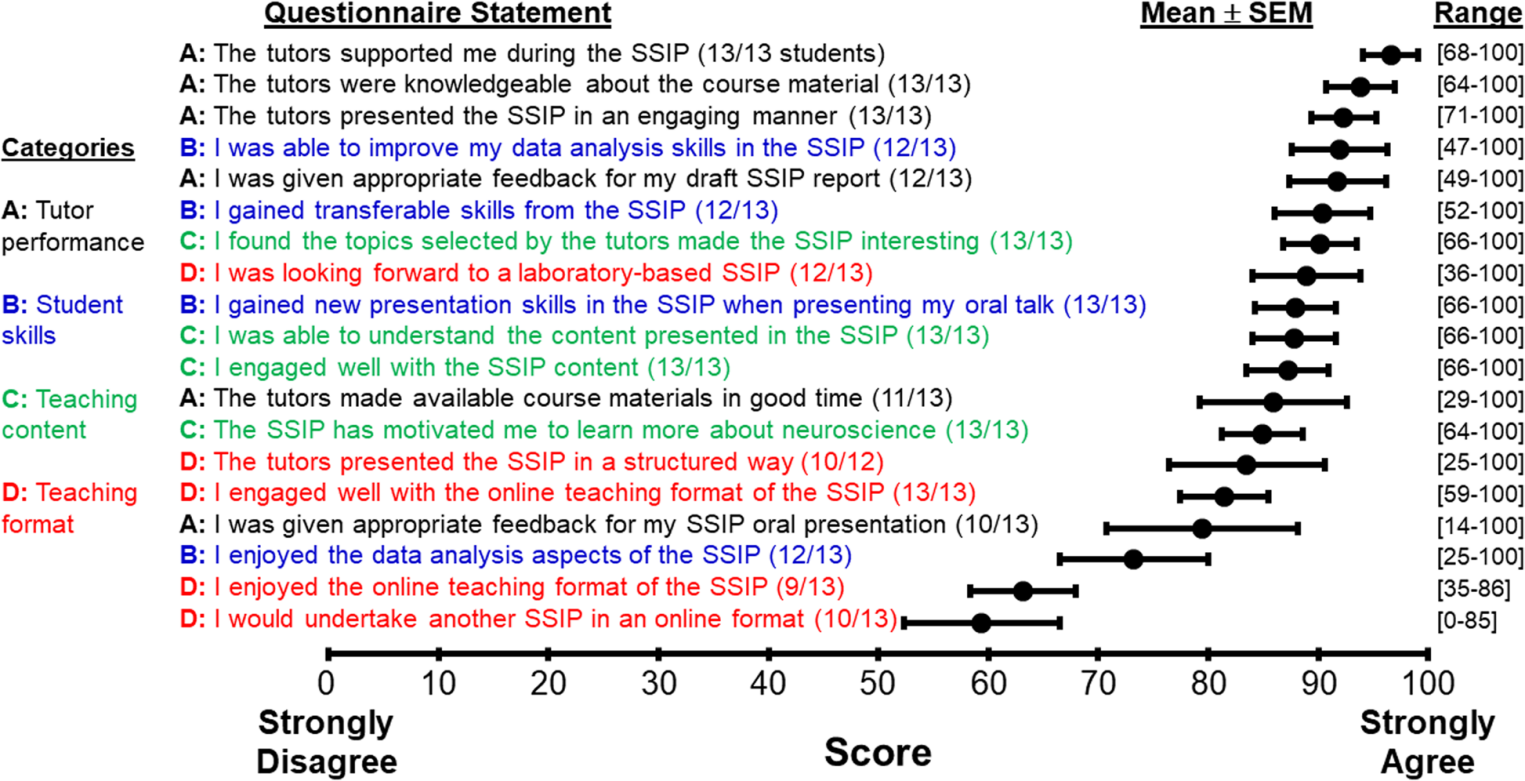
Questionnaire statements arranged in rank order from highest (strongly agree) to lowest (strongly disagree) scores. The filled black circles represent the mean ± standard error of the mean. Parentheses after each questionnaire statement give the number of students who scored that statement > 50 (range: neutral to strongly agree). Square brackets give the range of participant responses (minimum and maximum values)

Thirteen out of 15 students completed the questionnaire (87%); all who completed did so within two days of dissemination. Figure 1 shows student responses to 19 statements covering four categories: tutor performance, student skills, teaching content and teaching format. The mean scores for all the questionnaire statements exceeded 59/100 (although some individual responses were lower) indicating that most students agreed with the statements (Fig. 1).

Students most strongly agreed with statements associated within the tutor performance category (Fig. 1). The highest mean scores (*most strongly agree*) were given for statements related to the level of tutor support (96.6 ± 2.5), tutor knowledge of course material (93.9 ± 3.1), the ability of tutors to present material in an engaging manner (92.3 ± 2.9), and whether tutors gave appropriate feedback on reports (91.8 ± 4.4). Relatively lower mean agreement scores were given for tutors making course materials available in good time (85.9 ± 6.7), and provision of appropriate feedback on online oral presentations (79.5 ± 8.7).

Within the student skills category, students agreed on average that the online/active research course enabled them to improve data analysis skills (91.9 ± 4.4), gain transferable skills (90.4 ± 4.3), and oral presentation skills (87.9 ± 3.7). The average scores were lower when asked whether they enjoyed the data analysis aspects of the SSIP course online (73.2 ± 6.7).

For the teaching content category, on average students found the topics interesting (90.2 ± 3.3), understood the content (87.8 ± 3.8), engaged well with the content (87.2 ± 3.8) and were motivated to learn more about the topic (84.9 ± 3.7).

Students agreed least with statements within the online teaching format category, related to enjoyment of the online SSIP course (63.2 ± 4.8) and undertaking another SSIP online (59.4 ± 7.1). On average, students agreed that they were looking forward to a laboratory-based SSIP (88.9 ± 4.9), thinking at the point of allocation that it would have been held in-person and not in an online format. On average, students agreed that the SSIP teaching was presented in a structured way (83.5 ± 7.1) and they engaged well with the online teaching format (81.5 ± 4.0).

### Student *qualitative* perceptions of online course teaching

Table 1 lists five themes identified from free text student responses to the question, ‘*What did you enjoy most about the SSIP being taught online?*’ The most frequent theme for this question was related to convenience (54%), followed by use of online resources (23%) and use of screen sharing (15%). Other students enjoyed being able to experience research (8%) and communication/interaction online (8%). Table 2 lists three themes identified for the question ‘*What did you least like about the SSIP being taught online?*’ The most frequent theme for this question was related to communication/interaction (46%), followed by laboratory skills (31%) and engagement (23%). Table 3 lists four themes identified for the question ‘*What could be done to improve the SSIP being taught in an online format?*’ The most frequent theme for this question was related to resources (31%), followed by engagement (23%). A smaller number of students gave responses related to communication/interaction and laboratory skills (15% each). Table 4 lists six themes identified for the question ‘*What aspects of the SSIP would you prefer to be taught online …?*’ and six themes identified for ‘*… and which aspects would you prefer to be taught in person?*’. The most frequent themes for online teaching were related to content and data analysis (23% each), and for in-person teaching, the most frequent student responses were related to presentation skills (23%) and communication/interaction (15%).

**Table 1:** Themes identified from *student* responses to the question, ‘*What did you enjoy most about the SSIP being taught online?*’

**Themes**	**Student Responses** **(*n* = 13 max)**	**Example Quotes**
**Convenience**	7 (54%)	*“It allowed me to take notes easier during the sessions as I was sat at a desk rather than in a laboratory.”* *“It was easier it terms of not having to leave my room, I could do all my learning just by sitting at my desk.”*
**Resources**	3 (23%)	*“I liked that there was recordings of the meetings to refer back to and that all the resources we needed were in one place.”*
**Screen sharing**	2 (15%)	*“Our tutors used the teams functions (screen sharing) very effectively to enhance our learning.”*
**Research**	1 (8%)	*“Being able to see real-life research.”*
**Communication / Interaction**	1 (8%)	*“Being able to meet some Hull-based students.” [Note: this student is based on the York Campus]*

**Table 2:** Themes identified from *student* responses to the question, ‘*What did you least like about the SSIP being taught online?*’

**Themes**	**Student Responses** **(*n* = 13 max)**	**Example Quotes**
**Convenience**	6 (46%)	*“I didn’t get to meet my tutors and the other members in my group in person.”*
**Laboratory skills**	4 (31%)	*“I was looking forward to the practical elements in the lab which could not be done online.”*
**Engagement**	3 (23%)	*“Possibly a bit less engaged because it doesn’t feel as serious as if you were sat there in person paying attention.”*

**Table 3:** Themes identified from *student* responses to the question, ‘*What could be done to improve the SSIP being taught in an online format?*’

**Themes**	**Student Responses** **(*n* = 13 max)**	**Example Quotes**
**Resources**	4 (31%)	*“More resources provided and ongoing feedback.”*
**Engagement**	3 (23%)	*“Possibly put our cameras on because as nice as it is to not have them on, it’s easier to loose focus.”* *“Making the sessions only an hour/hour and a half long.”*
**Communication / Interaction**	2 (15%)	*“Try and fit in one or two sessions in person (when COVID allows) so we can engage more as a group.”*
**Laboratory skills**	2 (15%)	*“Possibly show us some of what we would have done in the lab.”*

**Table 4:** Themes identified from *student* responses to the question, ‘*Which aspects of the SSIP would you prefer to be taught online and which aspects would you prefer to be taught in person?*’

**Themes**	**Student Responses** **(*n* = 13 max)**	**Example Quotes**
**Online**		
**Content**	3 (23%)	*“I would prefer to be taught the content regarding the studies … online”*
**Data analysis**	3 (23%)	*“Aspects such as data analysis … best online.”*
**Feedback**	2 (15%)	*“Aspects such as … feedback best online.”*
**General preference**	2 (15%)	*“For this ssip I preferred all aspects online as I felt that it worked well.”*
**Presentation skills**	1 (8%)	*“… oral presentations.”*
**Academic writing**	1 (8%)	*“Aspects such as … report writing … best online.”*
**In-Person**		
**Presentation skills**	3 (23%)	*“I would’ve loved to have been able to do presentation skills and complete the presentation in person.”*
**Communication / Interaction**	2 (15%)	*“I would like to … do group tasks and introductions in person.”*
**Data analysis**	1 (8%)	*“Statistics would be beneficial being taught in person ….”*
**Feedback**	1 (8%)	*“I would like to … receive feedback … in person.”*
**General preference**	1 (8%)	*“I like learning about things in person.”*
**Laboratory skills**	1 (8%)	*“Lab work could be taught in person.”*

### Tutor *qualitative* perceptions of online course teaching

As there were only three tutors, we have included all their responses to the free text questions (Tables 5, 6, 7 and 8). The four themes identified for the question, ‘*What did you enjoy most about the SSIP being taught online?*’ were related to communication and interaction, collaborative teaching, convenience, and novelty (Table 5). Communication and interaction, laboratory skills and preparation time were the three themes identified for the question, ‘*What did you least like about the SSIP being taught online?*’ (Table 6). Only one theme, communication and interaction, was identified for the question, *‘What could be done to improve the SSIP being taught in an online format?’* (Table 7). Therefore, communication and interaction were a common theme throughout tutor responses to these three questions. Four themes were identified for preferences for online teaching (data analysis, academic writing, presentation skills and content) and two themes for in-person teaching (laboratory skills and general preference) (Table 8).

**Table 5:** Themes identified from *tutor* responses to the question, ‘*What did you enjoy most about the SSIP being taught online*?’

**Themes**	**Example Quotes**
**Communication / Interaction**	*“It is easy to ask a question/set a short task and then read the immediate response on the Chat feature in Teams.”* *“More flexible communication with the students.”*
**Collaborative teaching**	*“I enjoyed the pre and post-meeting sessions with the other course tutors. Such discussions were helpful and enabled tutors to know clearly what was expected from each tutor and the plan for the programme.”* *“Being able to teach collaboratively with tutors all over the UK. The SSIP definitely benefited from the diverse experience, viewpoints and teaching styles of the different collaborators. Although we had twice as many students, we were able to share the teaching and marking load and discuss all aspects of teaching throughout the course.”*
**Convenience**	*“Convenience in meeting the students for the sessions.”*
**Novelty**	*“It was interesting to have a new way of delivering SSIP sessions.”*

**Table 6:** Themes identified from *tutor* responses to the question, ‘*What did you least like about the SSIP being taught online*?’

**Themes**	**Example Quotes**
**Communication / Interaction**	*“Talking to a blank screen as the students typically would turn off their camera and microphone. Lack of student interaction and questions. Not being able to speak to the students informally.”* *“Lack of face-to-face communication.”* *“It was more difficult to get to know the students—they didn't always turn on their cameras during the group sessions. It was also more difficult to gauge the interest of the students and modify teaching accordingly, without the benefit of body language in person.”*
**Laboratory skills**	*“Not being able to teach face-to-face laboratory skills in a physical space.”* *“Lack of laboratory-based practice.”*
**Preparation time**	*“The amount of time spent preparing materials for online sessions felt much more involved than face-to-face.”*

**Table 7:** Themes identified from *tutor* responses to the question, ‘*What could be done to improve the SSIP being taught in an online format?*’

**Themes**	**Example Quotes**
**Communication / Interaction**	*“Although we tried to make sessions interactive, this wasn't always successful. Next time, I would modify sessions to incorporate more interactive elements, e.g. getting students to post their views in the chat or ask questions on camera. Also, shorten online sessions, ask them to keep their cameras on, or break it down into smaller groups or breakout rooms.”* *“Perhaps make some of the sessions more student led, which may increase their participation and interactions.”* *“More collaborative approach that may encourage students to be more interactive, visible and communicative (not being able to be reactive or inactive during the sessions).”*

**Table 8:** Themes identified from *tutor* responses to the question, ‘*Which aspects of the SSIP would you prefer to be taught online and which aspects would you prefer to be taught in person?*’

**Themes**	**Example Quotes**
**Online**	
**Data analysis**	*“… statistics could be taught in an online format.”* *“Analysis … would be online.”*
**Academic writing**	*“Writing skills … could be taught in an online format.”*
**Presentation skills**	*“…communication skills … could be taught in an online format.”*
**Content**	*“… tutorials would be online.”*
**In-Person**	
**Laboratory skills**	*“I prefer the laboratory sessions to be taught in-person.”* *“Practice-based activities like experimental procedures (EEG recordings) would be taught in-person.”* *“Laboratory experience, however, is best achieved in person, allowing students to be immersed in a lab setting, handling actual lab equipment, collecting data and especially interacting with each other.”*
**General preferences**	*“Teaching could be done online, but I feel students and tutors miss important unconscious cues and dynamics that facilitate learning that only happen in person. Students respond to passionate and enthusiastic teaching and this is much more difficult to achieve online.”*

### Comparison of academic assessment marks

**Fig. 2:**
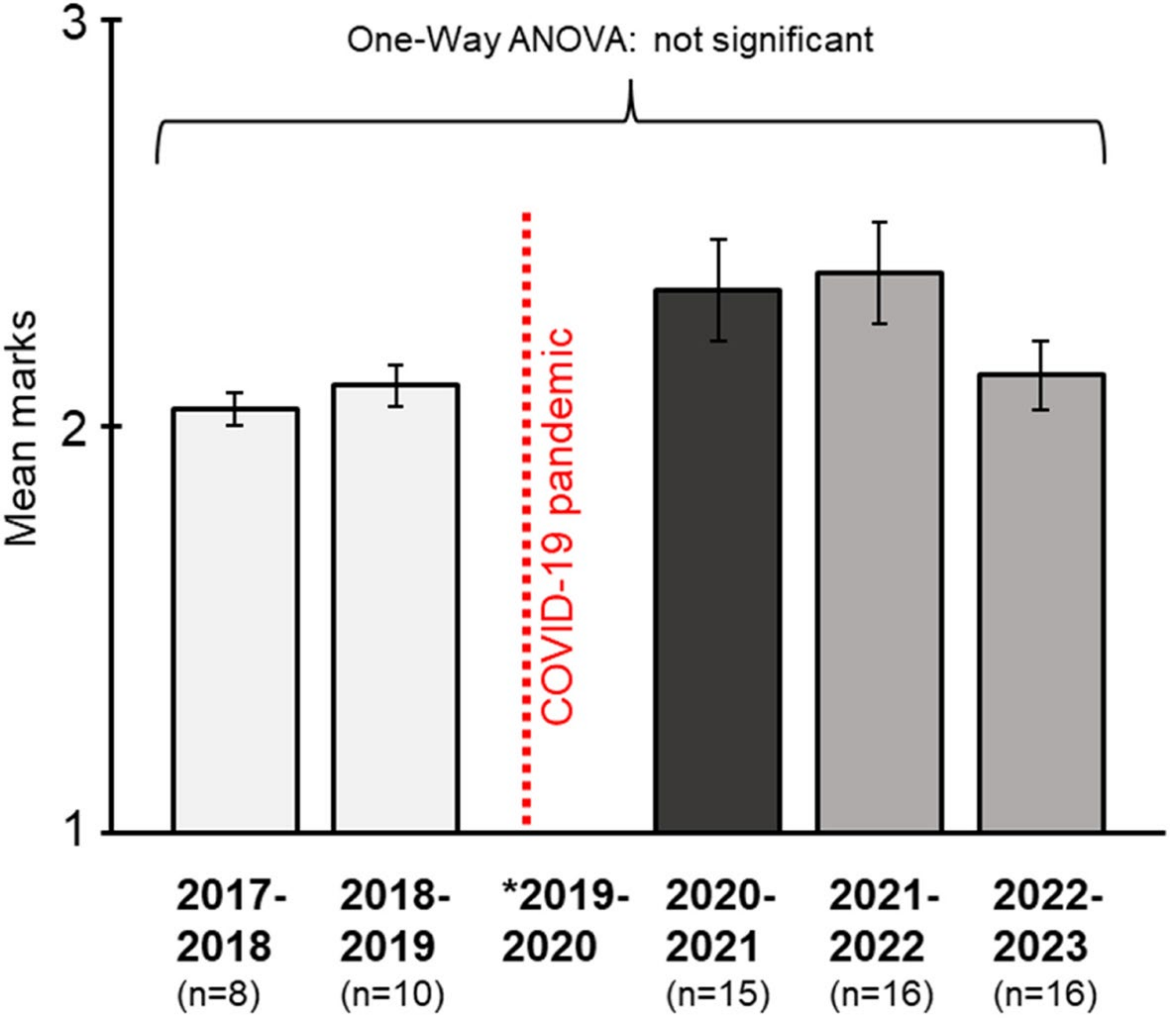
Plot showing the mean assessment marks for the SSIP teaching spanning the years 2017–2023. The vertical dotted line represents the start of the COVID-19 pandemic. A one-way ANOVA revealed no significant difference across the years (*p* > 0.05). *There were no SSIP assessments for the year 2019–2020. Marks: 1 = Fail, 2 = Pass, 3 = Excellent

Student attendance overall for the online/active research SSIP was 97.5%; out of eight mandatory teaching sessions for 15 students (8 sessions × 15 students = 120), a total of 117/120 attended with only three absences over the course. Assignment completion was 100%, and every student submitted their assignments within the deadline set. As an objective measure to determine whether learning outcomes were met during the year of the study (2020–2021), we compared summative assessment marks across five teaching years (2017–2023) which included two years prior to the onset of the COVID-19 pandemic and two years after the study year (Fig. 2). A one-way ANOVA revealed no significant difference for summative marks across all five years, F(4, 60) = 1.84, *p* = 0.133, eta squared = 0.109. All students achieved a Pass or Excellent mark with no Fails.

## Discussion

### Learning outcomes and developing student research skills

In this study we were interested in investigating student perceptions of a course delivered online which involved them in an active research study. The student responses to questionnaire statements indicate that despite major changes from in-person laboratory-based SSIP teaching to the fully online format using an active research study, students were largely positive about most aspects of the redesigned course delivered during the COVID-19 pandemic. Even though students agreed least with the statements related to enjoying the online teaching format, and whether they would undertake another SSIP online, the average agreement scores were still above 50%. While students expressed a preference for a laboratory-based course, they nonetheless reported that they developed valuable research skills from the online/active research course. Students agreed that they were able to acquire research skills, particularly related to data analysis, transferable skills, and giving an oral scientific presentation. However, they found the online data analysis component comparatively less enjoyable.

Assessments required students to demonstrate research skills gained, including researching and consolidating the relevant scientific literature, data analysis and visualisation, and interpretation of the results in the context of the field. In addition, students presented an overview of their selected topic based on their review of the literature, and generated a scientific report based on their data analysis, demonstrating their presentation and written research skills. SSIP summative assessment marks for the online/active research course in 2020–2021 were comparable to marks in earlier and later years, providing evidence that students achieved the learning outcomes and successfully acquired research skills.

### Benefits of using an active research study to engage and motivate students

Our survey results show that using an active research study led to high student engagement with the online SSIP content and motivated them to learn more about the topic. There are several reasons that could explain why our students found the online/active research SSIP course engaging and motivating. Firstly, the choice of topic, the effects of COVID-19 on memory, may have been of special interest because it was timely and of current global concern. Most students agreed (a score of ~ 90%) that the topic was interesting, and that the teaching content was understandable. One student stated that the most enjoyable part of the online teaching was *“Being able to see real-life research”.* Secondly, students had the opportunity to contribute to an ongoing research study by distributing the survey online to recruit participants and by performing preliminary data analyses. Thirdly**,** the small group size (15 students to three tutors, a student-staff ratio of 5:1) may have encouraged greater student-to-tutor and student-to-student interactions (approximately half the students were located in Hull and half in York). In a previous study, some UK medical students reported that small group sizes elevated student engagement [16]. Indeed, one student based in York reported that the most enjoyable aspect of the online teaching was, *“Being able to meet some Hull-based students.”* Fourthly, students may have been more engaged because they were especially pleased with tutor performance. Indeed, the three statements with highest agreement from the students were that they felt well supported by tutors, that tutors were knowledgeable about the course and presented material in an engaging manner. Given that tutors were invested in the active research study, this may have been reflected in their knowledge of and enthusiasm for the topic.

In line with our results, a study of first-year undergraduate medical students demonstrated that early experiences of successful engagement with authentic research practices increases subsequent motivation for research [21]. Advice given by Ommering et al. states the importance of providing students with authentic research experiences, in particular addressing authentic research questions of clinical importance where possible [22]. The active research study used in the current investigation was timely and clinically relevant given the impact of COVID-19 on cognition, particularly memory function [12]. Engaging medical students with authentic research experiences early in their career is a potential way to reverse the decline in the clinical academic (also called ‘physician scientist’) workforce [23].

Learning context is important. Embedding an active research study in research methods training aligns with the principles of situated learning [24]. Lave and Wenger describe how learners learn through legitimate peripheral participation and benefit from exposure to a community of practice [25]. Through small-group discussions with expert tutors and exposure to real-world data, the students in this study began to integrate into the research community (five of the students from this group have actively sought to continue their involvement with research post-SSIP). Indeed, in their ‘Twelve tips’ guide to encouraging student engagement in academic medicine, Lawson McLean et al. encourage involving students in the practicalities of research [26]. Involving students in authentic ongoing research has been shown to benefit students in other practical disciplines such as language translation, with the potential to enhance the proficiency of students both as researchers and as reflective practitioners [27].

### Benefits to research

Involving undergraduates had a direct benefit to our research study. For example, by distributing the research survey/memory quiz link to their networks, they aided in participant recruitment. In addition, they helped identify relevant references from the scientific literature and summarised them in their oral presentations. Another benefit of involving students was that they provided a diversity of perspectives, experiences and previously acquired skills to our research study. Based on these benefits, we recommend that educators consider involving undergraduate students in an active research study. Tutors may first need to consider the appropriateness of the research project for undergraduate teaching purposes. A second consideration is to ensure that ethical approval allows for student involvement in the research study, including aspects related to safety and confidentiality. Thirdly, the timing of the teaching sessions needs to be coordinated within the context of the research study, e.g., data collection. An alternative would be to involve students only in secondary data analysis, which would allow for greater flexibility. Overall, using an active research study not only advances student research skills, but can also bring value to the research project itself.

### Benefits of the online teaching format

In a study investigating online clinical medicine teaching, faculty members reported high satisfaction with student engagement levels and the quality of student interactions for the online technology-enhanced sessions but low satisfaction with the in-person traditional clinical sessions [28]. In our current study, student and tutor free text responses indicated that they particularly enjoyed the convenience of learning/working online, the availability and use of online resources and the use of online video technology (screen sharing). Because it was online, it meant it was easier for students and tutors to attend without the need to commute, which was important because attendance for the SSIP was mandatory. Student attendance for our online SSIP teaching was high (97.5%), which is similar to the 100% attendance reported by Kay & Pasarica in a study using online teaching in medical education where the attendance was also mandatory [28]. This study reported that 100% of their students completed the online assignments (*n* = 27), which aligns with the 100% assignment completion rate in our online SSIP teaching (*n* = 15). This shows that for mandatory online teaching sessions, attendance and assignment completion rates are high.

Another aspect of online teaching that students liked was the availability of online resources which they could view before or after online sessions. For example, we made online resources available including the documents associated with the research study, literature references and video recordings on how to analyse the data using statistics. Our approach to the online sessions was in line with recommendations outlined by Ohnigian and colleagues [29]. For example, we made use of the chat function and encouraged students to turn their cameras on to ask questions and interact with the tutors and other students. Some students mentioned that they enjoyed most the way tutors used screen sharing function in Teams to demonstrate specific concepts, such as data analysis.

From the tutors’ perspective, they enjoyed the novelty and collaborative aspects of online teaching. For example, one tutor stated that they enjoyed the pre- and post-meeting sessions with the other course tutors. Another tutor pointed out that a benefit of working online with tutors at different locations was to gain from their diverse perspectives and teaching styles. Although these positives are both possible with in-person teaching, the online format made collaboration across geographical boundaries easier. Another advantage of conducting the course online is that clinicians were able to contribute to one of our online teaching sessions, which would have been much more difficult to arrange in person due to their demanding schedules.

### Disadvantages of online teaching

There were aspects that students and tutors were less positive about the online teaching. Both students and tutors highlighted the lack of face-to-face interactions. Students were not able to meet tutors and other members of the group in person. Both students and tutors were concerned that the online format reduced student engagement. The lack of interaction with fellow students has been noted in a previous study which highlighted problems with student motivation, concentration and asking questions online [16]. Since the SSIP students often turned off their cameras and microphones, tutors also expressed concern that they were less engaged talking to a blank screen. Using a phenomenological approach, Schwenck & Pryor found that it was important for students to have cameras switched on rather than looking at a blank screen to feel engaged and connected [30]. Although there are several reasons why students do not turn their video cameras on, including it being considered the norm, and concerns about physical appearance or screen background, it may be possible to use strategies such as active learning techniques to encourage camera usage [31]. Cheung and colleagues found that students’ perceptions of online teaching were more favourable when video cameras were turned on so, although students are reluctant to do so, as educators, we should support students to turn their cameras on in sessions [32].

When students were asked which aspects of the SSIP they would prefer to be taught online and which aspects they would prefer to be taught in person, there was, in many cases, little agreement amongst students. For example, some students would prefer data analysis and statistics to be taught online whereas others would prefer these subjects to be taught in person. Similarly, some students think oral presentations should be done online, some think they should be done in person. This reflects the heterogenous nature of the student body and tutors should be mindful of this. Tutors could cater for the needs of a diverse group by offering multiple formats of engagement to increase accessibility. For instance, data analysis classes could alternate between classes being held online and in person.

Both students and tutors mentioned that they would have benefited from the experiences of learning and teaching in a physical laboratory space. One important point was that students were not able to develop laboratory skills that could best be learned using a hands-on, practical approach. One student captured this by stating, ‘*I was looking forward to the practical elements in the lab which could not be done online’.* Colthorpe & Ainscough similarly found that although students believe the online teaching to be helpful, the lack of in-person laboratory classes and face-to-face interactions negatively affected their learning experience [11]. In our study, two students suggested that a compromise could be to demonstrate online some of the practical skills that would normally be done in the laboratory. One limitation of our study is that as the Year 1 students started during the COVID-19 pandemic, they did not have any prior experience with in-person laboratory teaching within the medical curriculum. Therefore, they were not able to compare the SSIP teaching we delivered online with a face-to-face taught laboratory course. Moreover, since the SSIP teaching coincided with a national lockdown, this may have impacted on how well students engaged with the online course. Because students were mandated to stay at home, they could have seen the online SSIP teaching as one of their only opportunities to gain research experience and interact with students/tutors, which may have inclined them to respond more favourably to our questionnaire.

One tutor expressed concern that more time was needed to prepare materials for the online sessions compared to in person. Given that tutors had to become familiar with new online software and features to deliver online teaching, this will have increased their preparation time. In line with this, a survey of academics found that more time is needed to prepare for online teaching compared to on-campus teaching [33].

Students and tutors both suggested future improvements to the online SSIP teaching. For example, recommendations included making the online course more interactive, keeping cameras on, using breakout rooms and the chat feature more, incorporating student-led sessions and keeping sessions shorter. When students and tutors were asked which aspects they preferred to be taught online versus in person, several students and tutors suggested that content and data analysis could be taught online, while laboratory skills could be taught in person.

### Limitations

One limitation of the current study is the relatively small number of student (*n* = 13) and tutor (*n* = 3) participants. Ours is not the first study to consider the views of small numbers of medical students engaging with innovative teaching practices. For example, Margolin et al. considered the views of 13 medical students to make recommendations for online urologic education during the COVID-19 pandemic [34], and Blackard et al. piloted an online research training course with 27 medical students [35]. Our current study, which was undertaken in the context of small group teaching, provides data with initial indications that student perceptions were positive for teaching research skills online using an active research study during the COVID-19 pandemic. The hope is that our study encourages future studies using an active research study in larger cohorts across different medical schools and disciplines.

Both of the researchers (AURA and HAB) who coded the free text responses were involved in teaching and assessing the SSIP module and were investigators in the active research study. Whilst their familiarity with both the educational and research aspects of the project provided valuable context to the coding, we acknowledge that an independent researcher may have coded the free text comments differently.

Student engagement and motivation scores may have been affected by both the online teaching format and involvement in an active research study. We cannot disentangle the separate effects of each component in the current investigation. Each component would have to be evaluated in separate student groups, but such a study could lead to a lack of teaching parity across groups. A crossover design in which all students are exposed to each component consecutively could be another possible approach to extract the independent contributions of the online teaching versus the active research component.

## Conclusions

Taken together, our results indicate that a course can be delivered online using an active research study that will enable medical students to acquire research and scholarship skills, thereby fulfilling the *‘Clinical research and scholarship’* learning outcomes of the General Medical Council. More generally, this approach could be utilised as a model to deliver online teaching in other disciplines requiring the development of student research skills. It would enhance course accessibility and accommodate the needs of student groups who find it challenging to attend in-person courses such as students based outside the university, or those with physical disabilities or caring responsibilities. Moreover, online teaching with an active research component could encourage greater collaboration between instructors and researchers, as there would be fewer time and space constraints, thereby enriching the student and tutor experiences.

## Data Availability

The datasets used and/or analysed during the current study are available from the corresponding author on reasonable request.

## Abbreviations

SSIP: Scholarship and Special Interest Programme
SSC: Student-Selected Component
COVID-19: Coronavirus Disease 2019
VLE: Virtual Learning Environment
CORONA: COVID-19 Online Rapid Objective Neuro-Memory Assessment
URL: Uniform Resource Locator
